# Understanding Mechanical Response of Elastomeric Graphene Networks

**DOI:** 10.1038/srep13712

**Published:** 2015-09-08

**Authors:** Na Ni, Suelen Barg, Esther Garcia-Tunon, Felipe Macul Perez, Miriam Miranda, Cong Lu, Cecilia Mattevi, Eduardo Saiz

**Affiliations:** 1 Centre for Advanced Structural Ceramics, Department of Materials, Imperial College London, London SW7 2AZ, UK; 2 Department of Materials, Imperial College London, London SW7 2AZ, UK

## Abstract

Ultra-light porous networks based on nano-carbon materials (such as graphene or carbon nanotubes) have attracted increasing interest owing to their applications in wide fields from bioengineering to electrochemical devices. However, it is often difficult to translate the properties of nanomaterials to bulk three-dimensional networks with a control of their mechanical properties. In this work, we constructed elastomeric graphene porous networks with well-defined structures by freeze casting and thermal reduction, and investigated systematically the effect of key microstructural features. The porous networks made of large reduced graphene oxide flakes (>20 μm) are superelastic and exhibit high energy absorption, showing much enhanced mechanical properties than those with small flakes (<2 μm). A better restoration of the graphitic nature also has a considerable effect. In comparison, microstructural differences, such as the foam architecture or the cell size have smaller or negligible effect on the mechanical response. The recoverability and energy adsorption depend on density with the latter exhibiting a minimum due to the interplay between wall fracture and friction during deformation. These findings suggest that an improvement in the mechanical properties of porous graphene networks significantly depend on the engineering of the graphene flake that controls the property of the cell walls.

Ultra-light porous networks based on carbon nanomaterials (such as graphene or carbon nanotubes) have been attracting increasing interest in recent years owing to their multifunctional properties, which enable a wide range of applications from bioengineering to electrochemical devices^1^,^2^. They have been used as strain sensors^3^, oil absorbers^4^, membranes^5^, catalyst supports^6^, Joule heaters^7^, or in supercapacitors electrodes^8^ to name a few. In many of these applications the mechanical response (not only the strength and stiffness but also the ability to absorb energy or recover their shape after compression) plays a determining role in the performance of devices.

Because of the interest, many different approaches have been developed for the fabrication of graphene foams. They include the use of segregation reactions^9^, microwave synthesis^10^ or chemical vapour deposition (CVD) on a metallic foam that is subsequently eliminated to leave a free-standing graphene structure^11^. CVD can produce 3D materials that retain the properties of pristine graphene. However, these approaches are cumbersome or not well suited for mass production of macroscopic structures. An alternative is the use of wet processing techniques, most of which are based on suspensions made from chemically derived graphene (CMG). These include hydrothermal self-assembly^8^,^12^,^13^,^14^ sol-gel/carbonization^15^, sol-gel functionalization/polymerization^16^, lyofilization- microwave treatments^17^ and 3D printing^18^,^19^. Many wet processing technologies are based on the use of a template that is subsequently eliminated to generate porosity. This could be a soft template such as oil droplets in an emulsion^4^ or a hard template such as ceramic^20^ or polymer beads^21^. One version of this approach is based on the freezing of suspensions to use the ice crystals as a fugitive template for the formation of porosity^3^,^22^,^23^,^24^,^25^. Due to the versatility of the ice structure it is possible to create a variety of porous architectures, in particular it is possible to form layered materials that could exhibit very interesting mechanical responses. In general, wet processing approaches are simpler, enable some degree of structural control and are suited for mass production; they can also be used to form foams combining graphene with other materials^26^,^27^. However, they often require the use of additives and the electrical and mechanical response of CMG is intrinsically inferior to the properties of pristine graphene.

It is often difficult to control the mechanical properties of porous graphene constructs in a predictable way and there is still much work needed to fundamentally understand how the mechanical properties of these structures can be manipulated to address specific demands. As for any other foam, their mechanical response depends on their relative density, the pore geometry and the properties of the wall or struts^28^. In recent years several groups have used metals or ceramics to fabricate of ultralight, porous materials that retain stiffness and strength by manipulating these structural parameters^29^,^30^,^31^,^32^,^33^,^34^. It is becoming increasingly clear that the mechanical response depends on the interplay of chemistry and structure at multiple length scales from nano to macro levels but much work stills needs to be done in order to develop stronger and more reliable structures in practical dimensions. This is particularly complex when building structures from carbon nanomaterials such as carbon nanotubes or graphene. The translation of their unique intrinsic mechanical properties (high strength and stiffness) to practical macroscopic networks has been proved difficult. This is in part due to the difficulties associated to the fabrication of bulk materials with controlled architecture using particles with very high aspect ratio and to the manipulation of the interaction and assembly of individual particles (such as the graphene flakes) to build the internal walls of the structure.

In this work, we use chemically modified graphene to manipulate the chemistry and architecture (lamellar vs. cellular, pore size, density and graphene flake size) of 3D graphene-based porous networks fabricated by wet processing. The mechanical properties of these networks were investigated using compression tests and the energy absorption capability was carefully evaluated. The objective is to provide fundamental information needed to understand how the mechanical response of porous nanocarbon materials is defined by their structure at multiple length scales from nano to micro-levels. The final goal is to uncover basic data to guide the design and fabrication of structures with improved mechanical performance.

## Experimental

### Fabrication of graphene porous networks

**Figure 1:**
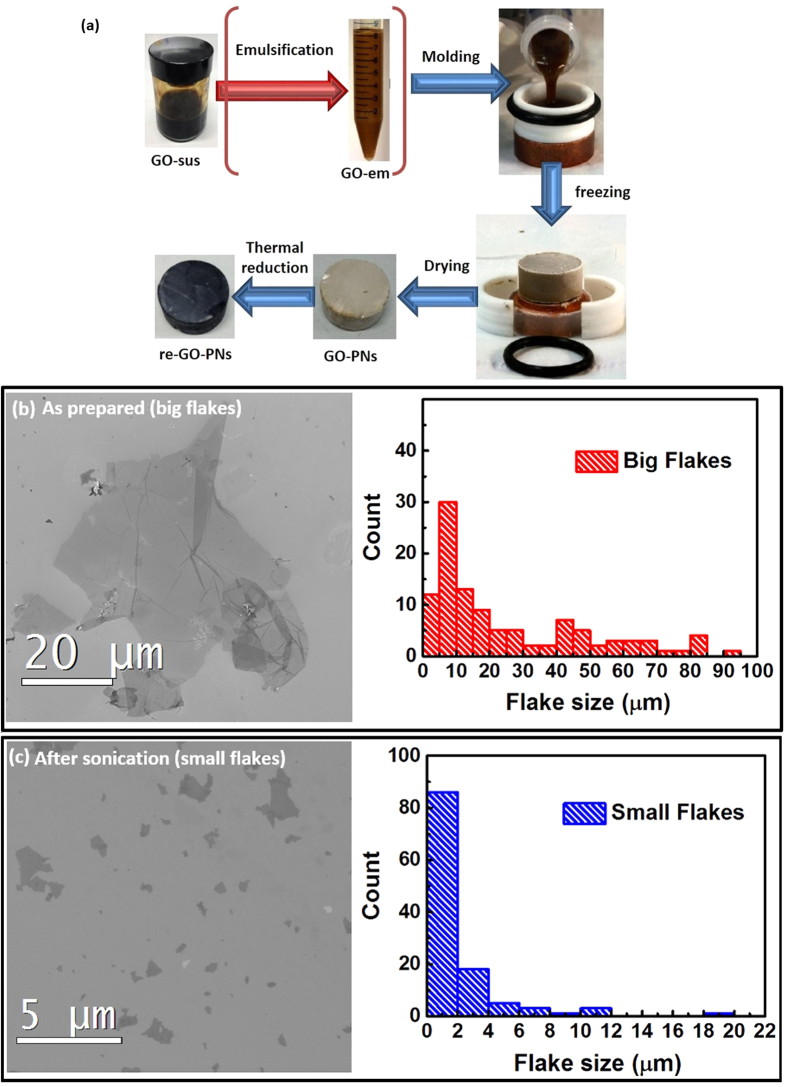
(**a**) Processing strategy of the porous networks. (**b**) and (**c**) are SEM micrographs and size distribution of GO flakes. (**b**) as prepared; (**c**) after sonication.

The processing routes are illustrated in Fig. 1a. GO aqueous suspensions (GO-sus) were prepared using the modified Hummers method^35^. Concentrated suspensions (2–20 mg/ml) were obtained by centrifugation and used for preparing GPNs with different microstructures. Organic additives (PVA:sucrose in a 1:1 fixed weight ratio, PVA in the format of 10% aqueous solution) were added to the suspensions, and the ratio of GO: additives was kept as 1:1 in weight. As a result, the concentrations of additives were between 0.2–2 wt% of the suspension. In the first route, the GO-sus was casted into cylindrical Teflon moulds placed on a copper cold finger. The suspension was then unidirectionally frozen by decreasing the temperature of the cold finger at a controlled rate varying between 1 to 10 K min^−1^ and subsequently freeze-dried (Freezone 4.5, Labconco Corporation) to eliminate the water. In an alternative route^4^, an extra emulsification step was carried out before freezing: the aqueous GO-sus were emulsified with a hydrophobic phase (toluene) in 1:3 or 3:1 volume ratio by hand shaking. The two phases (GO-sus and toluene) formed a homogeneous GO emulsion (GO-em) containing up to 75vol% of the toluene droplets. After freeze-drying bulk graphene oxide porous networks (GO-PNs) with cylindrical shape of ∼18 mm in diameter and ∼10 mm in height and densities between 3 and 15 mg cm^−3^ were obtained. Reduced graphene oxide porous networks (rGO-PNs) were obtained by thermal treatment of the GO-PNs at temperatures ranging between 473 to 1223 K for 20 min in a 10%H_2_/90%Ar atmosphere inside a tubular oven (Carbolite Furnaces).

In order to fabricate foams with reduced average GO flake size, ∼5 ml of a GO suspension (∼6 mg/ml) were subjected to sonication for 30 min in an ice bath using an ultrasonic tip (UP200S, Hielscher) at a power of 200 W, a frequency of 24 kHZ, an oscillation amplitude of 60% and a pulse of 0.5.

### Characterization

The GO flake size was measured using over 100 flakes by scanning electron microscopy (SEM, LEO Gemini 1525, operated at 5 kV). Samples were prepared by drop casting of diluted GO suspensions on silicon oxide substrates. The microstructure of the porous networks was investigated by SEM and aberration corrected transmission electron microscopy (TEM) (FEI Titan 80–300 S/TEM, operated at 80 kV). Foam cell size was measured from SEM micrographs using the principles described in ASTM D3576-04 (counting the number of cell walls which intersect a reference line). Raman measurements were carried out with a spectrometer (Renishaw RM2000 CCD) using a 514 nm laser excitation, laser power of 0.5 mW and 10 s integration time. The laser was focused onto the sample using a 50 times short working distance objective. Several spectra were collected from random locations on each sample. X-ray diffraction (XRD) patterns were collected using a PANanalytical® XRD X’Pert Pro diffractometer operated at 40 kV and 40 mA in the 2θ range 5°–35°, with a step size of 0.0334° and a count time at each step of 100 s.

The mechanical response of the networks was characterized by compression tests up to 80% strain using a universal mechanical testing machine (Z2.5, Zwick Roell, Germany) with a 2 kN load cell, in displacement controlled mode at strain rates ranging between 0.001 to 1 s^−1^. Samples with a diameter of ∼20 mm and a height of ∼10 mm were compressed along the freezing direction. Compression tests were also carried out *in situ* inside a HITACHI S-3400N SEM using a DEBEN microtest with a 300 N single leadscrew tensile module. Tests were performed in displacement controlled mode at 0.001–0.6 mm/s to record the morphological changes and elastic recovery of the structures. The electrical conductivity of rGO-PNs was measured using the four-point method by using a power supply unit (PSU) with constant input current of 0.17 A and a standard 4 channels bench multimeter to measure the voltage variation. For the electrical tests cylindrical samples of ∼1 cm in diameter and ∼2.3 cm in height were used and contacted to the circuit via copper electrodes.

## Results

### Microstructure of the porous networks

The as prepared GO suspension contains flakes with an average size of 25 μm (will be referred as “big flakes” hereafter). After subjecting to ultrasonication, the average flake size is reduced to 1.9 μm (will be referred as “small flakes” hereafter). SEM micrographs and size distribution of GO flakes are shown in Fig. 1b,c. Although the flake size has a wide distribution in both cases, the difference in size between the big and small flakes is significant.

**Figure 2:**
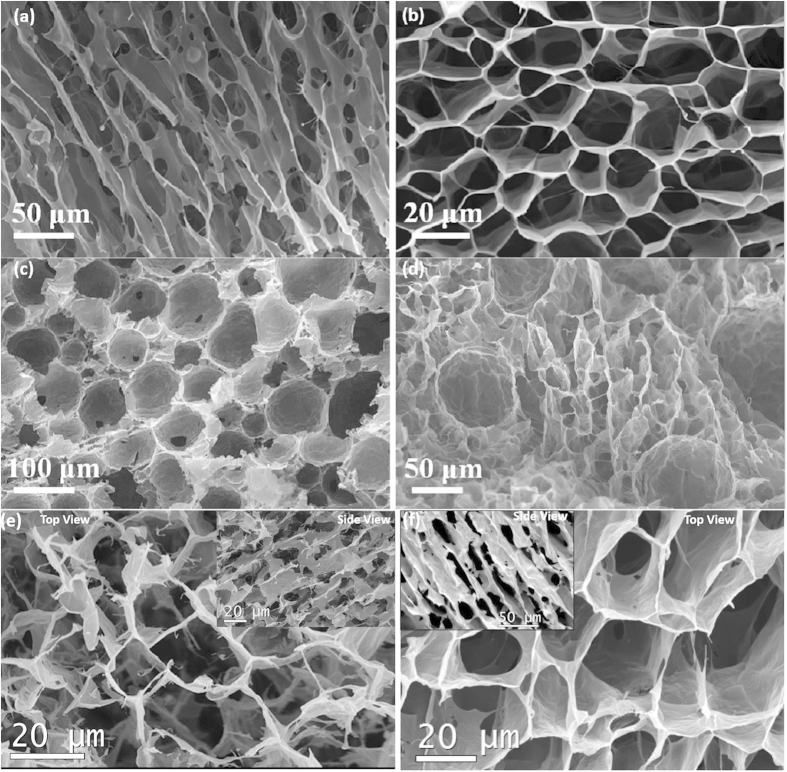
(**a**) Side (parallel to freezing direction) and (**b**) top view (perpendicular to freezing direction) of a GO-PN fabricated by freeze casting of GO-sus. The material exhibits a lamellar structure with a honeycomb-like cross sectional morphology. (**c**) Foam-like porous networks fabricated by using high concentrated oil-in-water emulsions (75 vol. %) and (**d**) hybrid foam-lamellar structure fabricated through the freeze casting of oil in water emulsions with low oil content (25 vol. %). (**e**) A lamellar GO-PN produced from GO-sus of same density (5 mg/ml) as those used for samples shown in (**a**,**b**), but using smaller GO flakes (<2 μm) than (**a**,**b**) (20–60 μm). (**f**) A rGO-PN network after the heat treatment at 1223K.

During freeze casting, ice crystals nucleate and grow in the aqueous phase while graphene oxide (GO) flakes are ejected from the moving ice front and align between the ice crystals, forming a continuous network. At the subsequent freeze drying step, the ice is sublimated, leaving behind a stable free-standing 3D GO-PN. The typical microstructures of GO-PNs are shown in Fig. 2. A *lamellar* structure with a honeycomb-like cross sectional morphology is observed for the samples fabricated without the emulsification step (Fig. 2a,b), similar to what have been reported for carbon based porous networks fabricated by freeze drying^3^,^24^,^25^.

A more complex structure was obtained for samples prepared from the GO-em. During freezing the emulsion oil droplets template the formation of spherical to polyhedral cells with ice forming within the aqueous continuous phase^4^. As a result, a predominantly isotropic *foam-like* structure is obtained from GO-em with a high oil content (75 vol. %) (Fig. 2c) and a hybrid *foam-lamellar* structure is formed from emulsions with a low oil content (25% vol.) (Fig. 2d). The porosity of the samples calculated based on the volumetric density of the samples^36^, ranges between 99.32% and 99.88%, which are similar to what have been reported in the literature for the graphene aerogel^17^ and elastomer^25^. These values have been calculated using the density of graphite as the theoretical density of the walls. However, the density of the walls can be lower as they are not formed by graphite but by the accumulation of CMG flakes.

The speed of the freezing front (manipulated by varying the cold finger cooling rate) can be used to manipulate the microstructure of the GO-PNs fabricated from GO-sus^37^. Lower speeds lead to GO porous networks with larger cells. Take a typical lamellar GO-PN with a density of ∼5 mg/cm^3^ as an example; the average cell size is 13.8 ± 3.4 μm (Fig. 2b) when the cold finger cooling rate is 10 K min^−1^ and increases to 46.6 ± 18.3 μm when the cooling rate is reduced to 1 K min^−1^ (Supplementary Fig. S1). A distribution of the cell size is also shown in Fig. S1. This tendency is similar to what was observed for graphene sponges fabricated by freeze drying of hydrothermally-reduced GO gels, where a higher solidification rate was found to reduce the mean pore size of the sponge^24^. For a similar density, a thicker cell wall is expected for the sample with larger cells, so in this work samples with larger cells are considered to have thicker cell walls. Reduction in the flake size was found to have an obvious effect on the microstructure of GO-PNs. The lamellar network produced with small flakes exhibits a much more fragmented structure (Fig. 2e).

**Figure 3:**
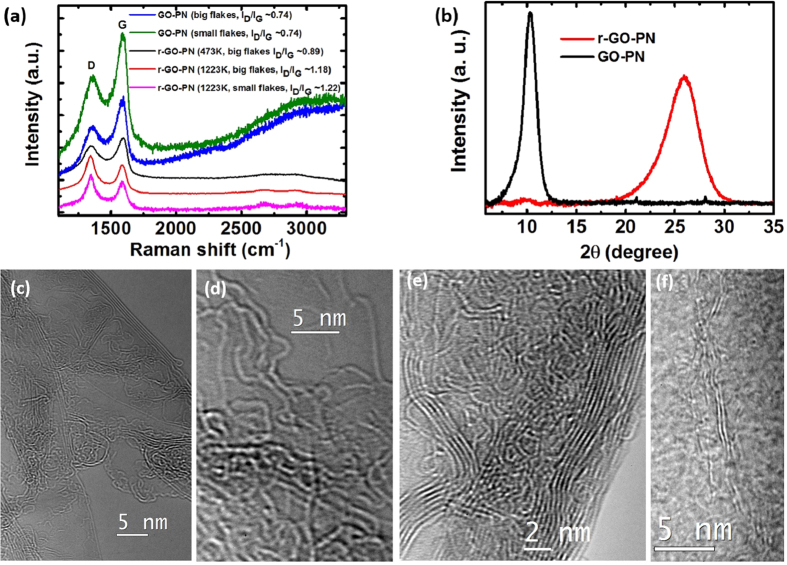
Characterization of the porous networks. (**a**) Raman spectra of the as prepared GO-PN and rGO-PN treated at 473 and 1223K. (**b**) XRD spectra of GO-sus, GO-PN and rGO-PN. (**c**–**f**) TEM observations. In (**c**) it is shown the presence of highly curved monolayer to few layer flakes in the rGO-PN. (**d**) In the high resolution phase contrast image of the edge of a single layer flake, in-plane carbon atoms are resolved and a variety of n-membered carbon rings can be seen. In (**e**) and (**f**) it is shown that the flakes composing the wall are entangled with each other.

Thermally reduced GO-PNs (rGO-PNs) retained the freeze casted microstructure (Fig. 2f). It has been shown in our previous work that most of the organic additives (∼91%) are removed during the heat treatment^4^. Raman spectra of the GO-PNs and rGO-PNs confirm the formation of predominantly crystalline rGO upon thermal reduction (Fig. 3a), indicated by the sharpening of both the D and G peaks and the enhancement of the 2D peak^38^,^39^. The spectra from samples treated at 1223 K exhibits more pronounced 2D peak than from that reduced at 473 K, suggesting that the crystallinity of the rGO increases with increasing treatment temperature, in agreement with our previous observations^4^. The intensity ratio between peak D and G (I_D_/I_G_) increases from ∼0.7 for GO-PNs, to ∼0.9 for rGO-PNs reduced at 473K and to ∼1.2 for rGO-PNs reduced at 1223K, corresponding to a decrease in the defect concentration as expected for carbon material with relatively high defect contents^38^,^40^. It is noted that the I_D_/I_G_ ratio for small flake samples is very similar to that for big flake samples, in both unreduced and reduced conditions, which indicates a similar atomic defect concentration in both samples. X-ray diffraction (XRD) patterns (Fig. 3b) provide further evidence on the removal of major oxygen-containing groups: the d-spacing of GO flakes in suspension is ∼0.83 nm and decreased to ∼0.34 nm in the rGO-PN, which is the same as the graphite interlayer distance. TEM analysis of the rGO-PN samples treated at 1223 K (Fig. 3c–f) reveals that the walls of the porous structure is composed of single, few-layer to multi-layer (up to ∼15 layers) graphene flakes having an interlayer spacing measured to be ∼0.35 nm, in good agreement with the spacing calculated from XRD. TEM micrographs (Fig. 3d) also reveal that the rGO flakes are highly curved with the presence of a variety of n-membered carbon rings, which is expected for rGO with a considerable defect density as indicated by the Raman spectra. It is noted that the flakes that compose the wall are entangled with each other (Fig. 3e,f).

### Mechanical response

Unless it is noted specifically, the results described in subsequent sections are for rGO-PNs produced under the most standard conditions in this work, i.e. use of big GO flakes, cold finger cooling rate of 10 K min^−1^ during freeze casting and subsequent heat treatment at 1223 K in 10%H_2_/90%Ar.

**Figure 4:**
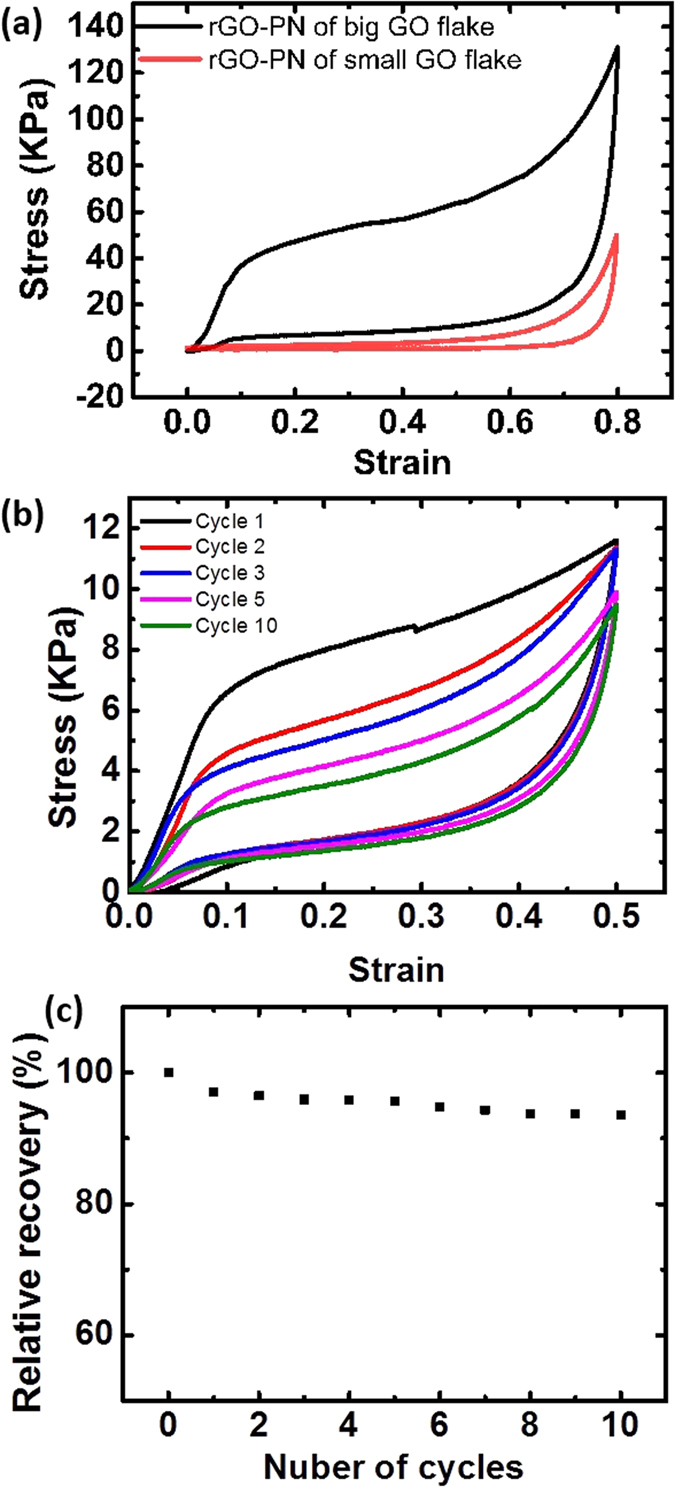
Typical compressive properties of the porous networks. (**a**) Compressive stress-strain curves tested at the maximum strain of 80% for two rGO-PN samples of similar density (ρ ∼ 11 mg/cm^3^) produced using big or small GO flakes. (**b**) The stress-strain curve for a rGO-PN (ρ ∼ 4.5 mg/cm^3^) tested at the maximum strain of 50% for 10 cycles. The strain rate for these test were 0.001 s^−1^. (**c**) The relative recovery after 50% strain for a rGO-PN with ρ ∼ 4.5 mg/cm^3^. Unless it is stated specifically the porous networks were produced using big GO-flakes with a cold finger cooling rate of 10 K min^−1^ during ice templating followed by heat treatment at 1223 K.

The porous networks show different behaviour under compression depending on their density^4^. In this work we mainly investigate the mechanical response of elastomeric rGO-PNs with densities between 1.5 to 12 mg/cm^3^, and an example is shown in Fig. 4a. Upon compression to a strain (ε) of 0.8, the stress-strain curve shows first a predominantly linear elastic region that can be associated to cell wall bending^41^, followed by a change in the slope that can be regarded as “elastic collapse” at strains typically <10% and a plateau region with a gradual increase in slope up to strains of ∼0.6. Finally a “densification” stage characterized by rapidly increasing stress with strain is observed. Overall, this behaviour resembles more an elastomeric foam where the elastic collapse is determined by buckling of the walls^41^. *In situ* SEM observation during compression of a lamellar network appears to confirm this collapse mechanism (Supplementary movie S1). The porous network fails via propagation of wall buckling at preferred locations (where the local stress field is maximized by local orientation of the wall). The mechanism for the collapse of the foam-like structure is similar with bending and buckling of the cell walls taking place at preferred stress locations (Supplementary movie S2). Multiple buckling processes, similar to what has been observed in carbon nanotube bundles^42^, may happen subsequently in different locations before the densification stage. This explains the small slope in the plateau region.

**Figure 5:**
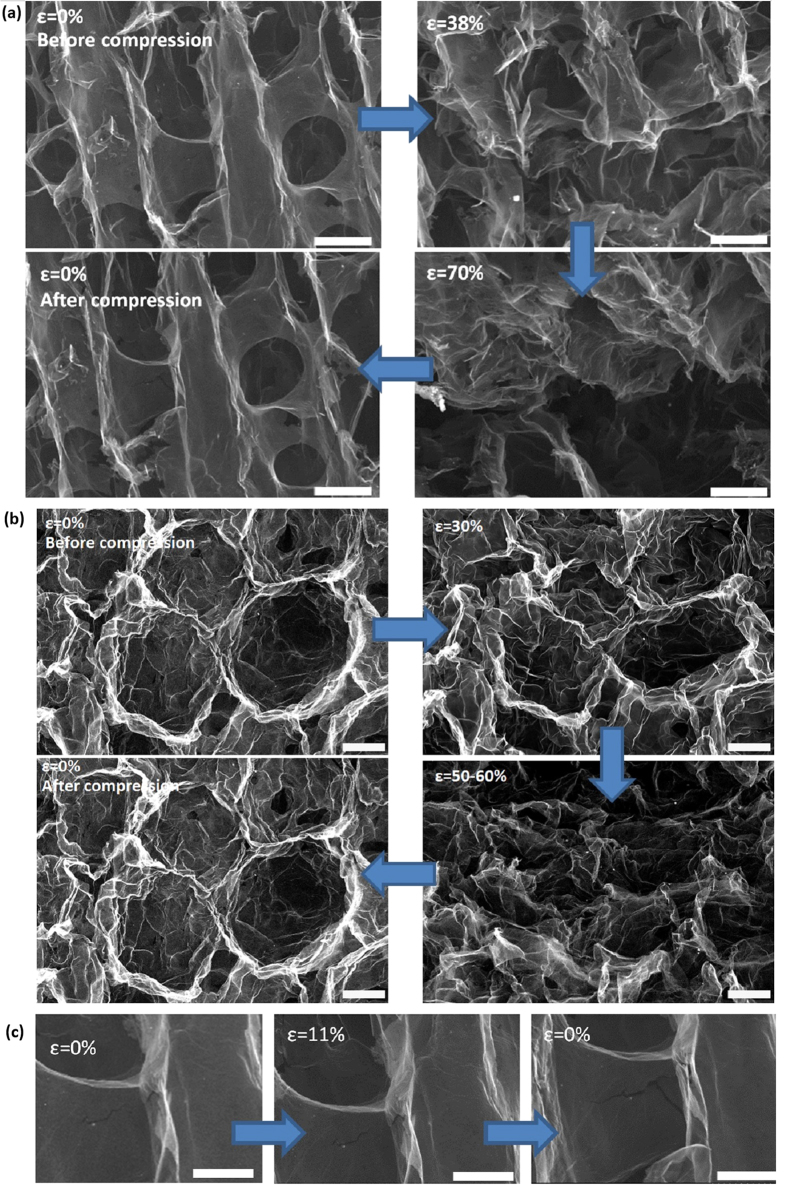
*In situ* high-resolution SEM images showing the microstructural change at different strain levels during the deformation for rGO-PNs with (**a**) lamellar structure and (**b**) cellular structure. Scale bar: 20 μm. (**c**) Evolution of a pre-exist defect during deformation. Scale bar: 10 μm.

The porous networks can recover its original volume with little macroscopic permanent deformation after being subjected to high compressive strains of up to 0.8 or after compressive cycling (Fig. 4b,c). The materials retain over 82% of their maximum stress after the cyclic compression. Large recoverability (<3% permanent deformation after 50% strain) was observed for samples with densities as low as ∼2.6 mg/cm^3^. While a direct comparison is difficult as the measured values are influenced by the foam density and testing conditions, this superelastic behaviour appears to be comparable or superior to other porous carbon structures^3^,^17^,^24^,^25^. The microstructural changes of the porous structure during compression can be followed by *in-situ* SEM (Fig. 5a,b). The microstructure recovers almost completely after loading is removed even after high deformations. On the other hand, propagation of pre-existing defects such as micro-cracks (Fig. 5c) and wrinkling of the cell walls (Figure S9b and c in reference 4) are responsible for the small non-recoverable deformation and the observed decrease in mechanical properties.

**Figure 6:**
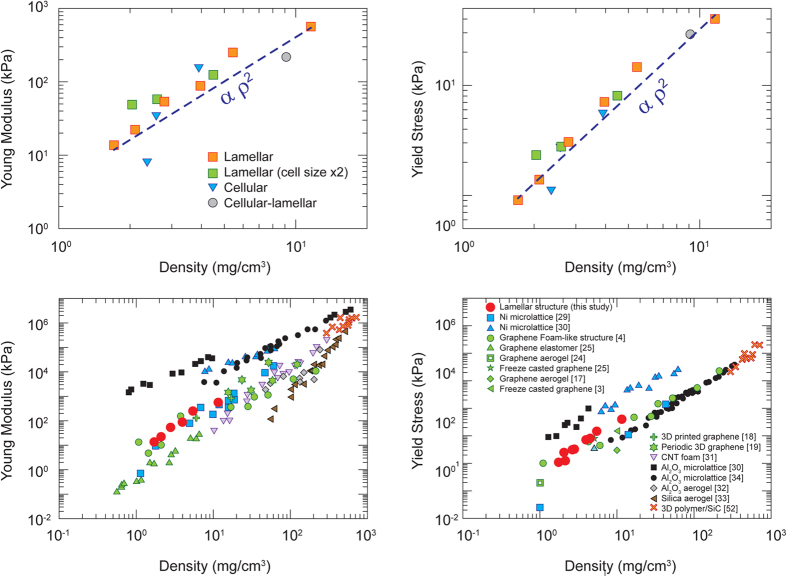
(**a**) Compressive modulus and (**b**) yield stress (collapse stress) of the rGO-PNs. (**c**) and (**d**) Comparison of the compressive modulus and yield stress with other porous networks. As noted in the main text, the lamellar structure has a cell size of ∼15 μm and the size is approximately doubled by decreasing the cooling rate from 10 K min^−1^ to 1 K min^−1^ for the sample labelled as “Lamellar (cell size × 2)”.

The compressive modulus and elastic collapse stress (yield stress) of the networks were estimated from the stress-strain curves as shown in Fig. 4a. The behaviour of both properties as a function of sample density and microstructural parameters studied (lamellar vs. foam-like, and increase in pore size in the lamellar network) are shown in Fig. 6a,b. As expected, both increase with increasing sample density; on the other hand, difference in the mechanical properties was small as a result of the variations in architecture (lamellar vs cellular) and pore size, although it appears that the modulus of sample with bigger pores is slightly higher.

**Figure 7:**
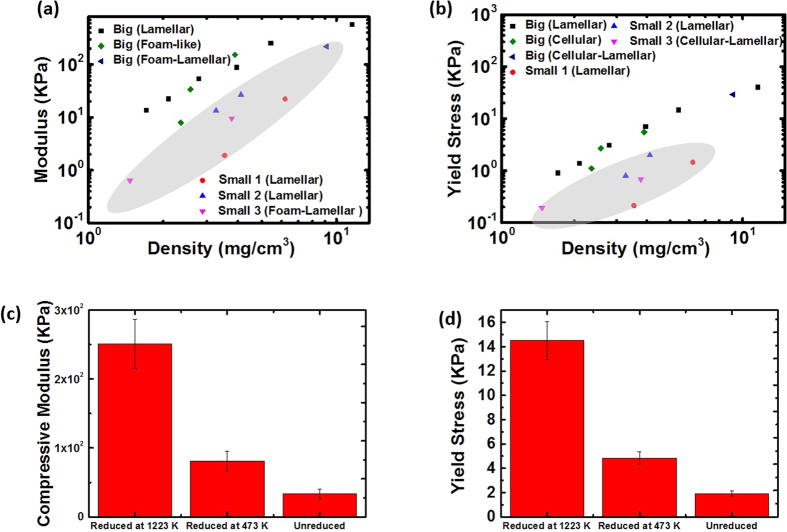
Effect of GO flake size (a,b) and heat treatment temperature (c,d) on the compressive modulus and the yield stress of the porous networks. In (**a**,**b**), data points from the small flake samples are shadowed. In (**c**) and (**d**), the densities of the tested samples are all ∼5 mg/cm^3^. In order to keep similar GO concentration in the starting solution, the unreduced GO-PN samples were produced without the addition of the binder.

The size of the building GO flakes plays a very important role in the final porous network properties. As clearly shown in Fig. 4a, the porous networks produced from small GO flakes are much weaker and exhibit no recovery ability after severe deformation. The effect can be more clearly seen in Fig. 7a,b. Independent of the microstructure (lamellar vs. foam-like), both the compressive modulus and the yield stress are significantly lower for the porous networks fabricated with small GO flakes. Taking into account the scatter of the data, the modulus and strength of the foams build using small flakes are one order of magnitude lower than those built from large flakes. The scattering in the data is larger in the case of samples with small flakes, likely from the variability in the distribution of defects in the wall.

The mechanical properties of the networks are also compared between samples that have similar final density (∼5 mg/cm^3^) but were subjected to different reduction conditions (Fig. 7c,d). Apparently, the reduction process enhances both the compressive modulus and the yield stress, and a higher reduction temperature has a greater effect. Similar enhancement in the mechanical properties by increasing the annealing temperature up to 473 K was also found for graphene paper^43^.

### Energy absorption

**Figure 8:**
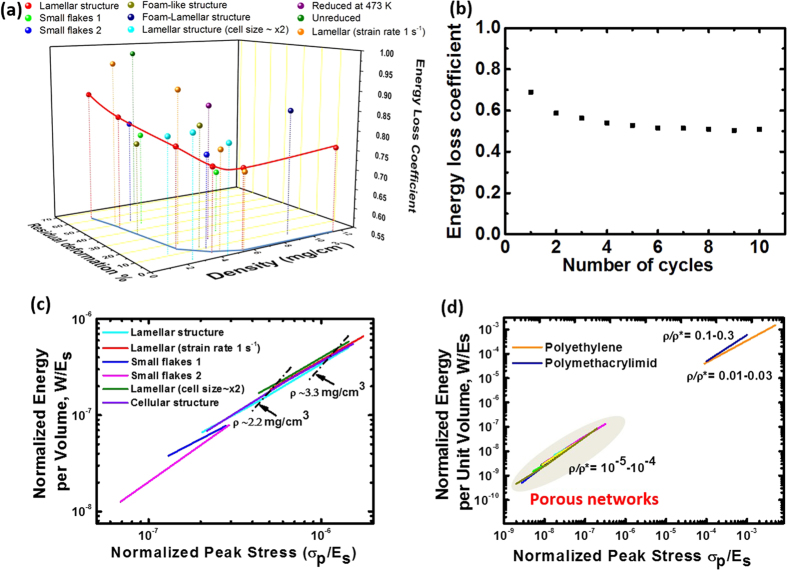
Energy loss coefficients of porous networks (**a**) produced with different conditions and (**b**) under a cyclic compressive test. (**c**) Energy-absorption diagrams for the porous networks produced and tested at different conditions. Parallel black dash-dot lines (arrowed) connect data for samples with same density but obtained at different strain rates, giving a family of lines of constant sample density. (**d**) Comparison between the porous networks and polymer foams in the energy-absorption diagram. Unless specified, the fabrication and testing conditions are: big GO flakes, lamellar structure, 10 K min^−1^ cold finger cooling rate during freeze casting, heat treatment at 1223 K and compressive test at strain rate of 0.001 s^−1^. As noted in the main text, the lamellar structure has a cell size of ∼15 μm and the size is approximately doubled by decreasing the cooling rate from 10 K min^−1^ to 1 K min^−1^ for the sample labelled as “Lamellar (cell size × 2)”.

Energy loss coefficients were calculated by taking the ratio between the energy dissipated within the materials and the work done by compression. Their variation with the density and permanent deformation is shown in Fig. 8a. Samples with densities >∼2–3 mg/cm^3^ are superelastic and their energy loss coefficient is as high as 0.84 at a density of 2.5 mg/cm^3^ for samples with larger GO flakes and thicker walls (larger cells). Furthermore, a good cycling performance is maintained, stabilizing the coefficient values around 0.55 after the first four compression cycles (Fig. 8b). The permanent deformation caused by the compression test decreases with increasing density as a result of increasing structural integrity (indicated by the blue solid line in Fig. 8a). On the other hand, the dependence of energy loss coefficient with density shows the presence of a minimum (indicated by the red solid line in Fig. 8a). The effect of strain rate on the energy dissipation efficiency also appears to depend on the sample density (or inherently the recoverability of the sample). At low densities when the structure does not recover, an increase in strain rate by 3 orders of magnitude (from 0.001 s^−1^ to 1 s^−1^) results in a higher amount of permanent deformation. At high densities the strain rate shows little effect on the amount of permanent deformation.

In general porous networks with larger cell sizes exhibit higher recoverability and energy loss coefficients. Networks produced from small flakes exhibit much larger permanent deformation at similar densities and lower energy loss coefficients when compared to samples with larger flakes.

### Discussion

The use of controlled directional freezing results in graphene networks with a well-organized structure and long- range order template by the ice crystals^37^,^44^. As expected for materials prepared from GO-sus the speed of the ice front and the concentration of the suspension determine the pore (cell) sizes with larger pores for lower speeds. Organic additives can influence the architecture and properties of the networks. As discussed in our previous work^4^, PVA changes the wettability and surface activity of GO; sucrose helps to hold the structure together and affects the shape of the ice crystals formed during freezing and therefore determining the topography of the cell walls. PVA is also known to act as a ‘cross-linking’ agent for GO^45^,^46^ and we have previously shown that addition of PVA and sucrose can affect density and rGO crystallinity^4^. In this paper all the samples were prepared using additives and we focus on the effect of other microstructural parameters.

The high elasticity and mechanical robustness of rGO porous materials has been attributed to both the degree of ordering at the microstructural level and the good structural alignment at the nanoscale (tight packing of rGO flakes in the walls)^25^. However, some rGO aerogels showing high recoverable deformation appear to have quite random microstructures^17^. The entanglement between the flakes in the cell walls is expected to provide strength and contribute to the superelasticity. Our results show that the size of the starting GO flakes plays a very important role in the final properties. Porous networks produced from small GO flakes exhibit much worse mechanical behaviour (Figs 4a and 7a,b). Fundamentally, this size effect can be related to the local packing of the flakes. For very small flakes it is more difficult to align and form a continuous tight-packed wall at long range during the freezing process, resulting in a high density of microscopic defects such as interflake pores (Fig. 2e) and reduced wall modulus and strength. For samples with very low densities, similar effects should be expected, which leads to the loss of superelasticity.

The compressive modulus of our superelastic networks can be tuned over 2 orders of magnitude^4^. The materials prepared using large flakes appear to be stiffer than previously reported porous carbon materials, especially at low densities (below 3 mg/cm^3^) (Fig. 6c). The yield strengths are also superior to other carbon foams reported in the literature and even comparable to some ceramic and metallic micro lattices of similar densities (Fig. 6d). The modulus was found to scale with the density as ∼ρ^2^, which would be expected for an open cell porous structure^41^. This indicates that the networks exhibit mechanical properties that resemble an open cell structure due to the openings of the cell walls.

For an open cell foam the young modulus, E, can be approximated by


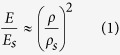


where E_s_ and ρ_s_ are the Young modulus and density of the wall respectively and ρ is the density of the foam^28^. As shown previously, the networks collapse by buckling of the wall (supplementary movie S1). Considering this mechanism, the yield stress, σ_el_, is expected to be expressed by the following equation:


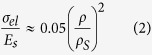


Taking into account that the relative densities range between 0.001–0.01, the wall modulus and stress calculated using equations (1) and (2) are consistent and of the order of ∼10 GPa. The modulus value is significantly lower than that of few-layer rGO (∼0.25 TPa^47^) but of the same order of magnitude to that of graphene paper (∼40GPa)^43^. Therefore the results support the idea of walls formed by the stacking of entangled graphene flakes held together by van der Walls type of forces (Fig. 3), similar to graphene paper.

Independent of the microstructure (lamellar vs. foam-like), both the compressive modulus and the yield stress are significantly lower for the networks fabricated with small GO flakes. As the modulus and strength of the foams build using small flakes are one order of magnitude lower than those built from large flakes, it is also suggested from equations (1) and (2) that the wall modulus of the sample made of small flakes is one order of magnitude lower. The electric conductivities of the networks prepared from large flakes are also one order of magnitude larger than that from small flakes (Supplementary Fig. S2). The increase in the electrical conductivity can be explained as a result of the overall lower contact resistance between larger flakes, similar to what has been reported for graphene films^48^. All the results suggest that the size of the building blocks (the GO flakes) plays a fundamental role in controlling both mechanical and electrical properties of graphene porous networks by determining the microstructure, in that larger flakes promote the formation of defect-free walls. The reduction process enhances both the compressive modulus and the yield stress, and a higher reduction temperature has a greater effect. Increasing the annealing temperature up to 473 K was also found to enhance the mechanical properties of graphene paper^43^. According to equation (1) and (2) this has to be related to an increase in the Young modulus of the cell wall, correlated to a recovery of the graphitic nature as can be seen from the Raman data (Fig. 4a). In the case of graphene paper the enhancement has been attributed to the better ordering and enhanced interlayer contact of graphene flakes after annealing. This seems consistent with the reduction in interlayer distance observed by XRD and TEM. The data therefore suggest that a less defective structure at the atomic level, resembling more to that of the ideal graphene, is crucial for improving the mechanical performance at the macro-scale.

The energy loss coefficient of the porous network is higher than those reported for graphene elastomers (0.825 at a higher density of 5.1 mg/cm^3^)^25^, graphene aerogels (0.75)^3^, foam-like CNT films (∼0.64)^49^ and Ni micro lattices (0.77)^29^, and almost as high as carbon nanotube bundles with much higher densities of ∼80 mg/cm^3^ (∼0.8–0.9)^42^. As suggested before^25^, the responsible mechanisms for the high energy loss coefficient could include the intra and inter wall van der Waals adhesion and friction during deformation and de-binding of the cell walls in order to recover their nearly original configuration upon unloading. For our networks this is supported by the observation of an increasing stress relaxation at increasing strain, especially above the yielding strain (ε ∼ 10%), as shown in Supplementary Fig. S3. These mechanisms are expected to be maximized due to the curved nature of the rGO flakes and their tight packing and entanglement in the cell wall (Fig. 3).

The presence of the minimum in the energy loss co-efficiency vs. density curve is a result of interplay of different energy dissipation mechanisms taking place in the porous network. At high densities (4 to 12 mg/cm^3^), the intra- and inter cell walls interactions are expected to increase with density, leading to increased heat dissipation due to van der Waals adhesion and friction resulting in higher energy loss coefficients. At low densities (1 to 4 mg/cm^3^), in addition to the above mentioned mechanisms, energy dissipation takes place through unrecoverable wall fracture in a way that the lighter the materials more dominant this mechanism becomes. As a result the energy loss coefficient increases towards lower densities. Similar energy absorption mechanisms as for samples with low densities can be present in samples without heat treatment or heat-treated at lower temperatures, as they also exhibit non recoverable deformation at densities as high as 5 mg/cm^3^ and high energy loss coefficient.

The porous networks with larger cell sizes exhibit higher recoverability and energy loss coefficients. For materials with similar densities, larger cells mean thicker walls that are able to provide better recovery and that more energy dissipation due to friction between flakes could be expected. The slightly lower energy loss coefficients of materials made from smaller flakes suggests that energy dissipation by interflake interaction in the wall is reduced in small flake samples. The observed dependence on the strain rate shows a similar tendency to what was reported for graphene elastomers^25^ and for some dense carbon nanotube structures^50^,^51^, while different from the behavior of carbon nanotube bundles^42^ where permanent deformation is reduced at higher strain rates.

For energy absorption applications, often the requirement is to absorb the kinetic energy while keeping the peak force below some limit. For a given material/structure, there is an optimum density at which an equivalent amount of energy can be absorbed at a lowest stress. Higher or lower densities lead to the required amount of energy absorbed at higher stress levels. We have develop energy-absorption diagrams^28^ for rGO-PNs fabricated under various conditions using the stress-strain curves of samples with different densities, and the results are shown in Fig. 8c,d, where *W* is the absorbed energy at a particular peak stress *σ*_*p*_ derived from the stress-strain curve, and both *W* and *σ*_*p*_ are normalized by the estimated modulus of the cell wall in our r-GO-PNs (*E*_*s*,_ ∼ 10 GPa). The principle and details of the procedure are described in the supplementary information and Supplementary Fig. S4. Each solid line in Fig. 8c,d and Supplementary Fig. S4 represents the optimum adsorption capability for the material of different densities under given conditions (allowable stress and expected strain-rates). In general, the samples behaviour can be described by the relationship 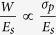, which is a master line expected for all low density elastomeric open cell foams independent of the material^28^. This explains the similarity of the lines for our porous networks with different microstructures (lamellar vs. foam-like) and confirms that the foams have an elastomeric behaviour.

The separation of the lines at different strain rates is expected as *E*_*s*_ is known to be dependent on the strain rates^28^, while we have used a single value (estimated modulus of the cell wall from our compression experiments, ∼10 GPa). In light of this, the separation of the lines corresponding to samples built from big or small flakes or with different cell sizes appear to suggest that the real modulus of the cell wall materials, which is different from the ideal graphene sheet, has a dependence on the rGO flake size and the thickness of the cell walls. A smaller *E*_*s*_ would have to be used to shift the line for the networks produced with small flakes upwards to obtain the master curve, indicating that the effective *E*_*s*_ of the network wall is smaller when it is composed of smaller flakes. Similarly, the envelope line shifted towards higher *W/E*_*s*_ values suggests a higher effective *E*_*s*_ for thicker walls. These results seem to agree with our measurements of the compressive modulus of the foams (Fig. 6a).

As expected for a master curve in the energy-absorption diagram, the behaviour of the rGO-PNs is aligned with other elastomeric open cell foams such as polyethylene (Fig. 8d) and slightly misaligned with plastic foams such as polymethacrylimid where the energy absorption dependence on the peak stress is expected to be slightly different. The fact that the data aligns with other elastomeric foams also suggests that the modulus we measured for the walls using compression is roughly right. Nevertheless, rGO-PNs can still offer measurable energy adsorption at much lower densities than that of conventional polymers foams combined with electrical conductivity and high recoverability. Moreover, their pore size is much smaller than in conventional porous polymer or ceramic foams with similar densities, which is of high interest for certain applications such as filtering membranes and catalysis.

## Conclusions

Elastomeric graphene porous networks with highly ordered lamellar structures were obtained by unidirectional freeze casting of GO water-based suspensions followed by thermal reduction. The microscopic structure was predictably controlled by a systematic change of the processing conditions: the size of the GO flakes, the incorporation of an emulsification step, the freezing rate and the thermal treatment temperature. The porous networks made of large rGO flakes (>20 μm) are superelastic and exhibit high energy absorption capability, showing much enhanced mechanical and electrical properties than those with small flake size (<2 μm). A better restoration of the graphitic nature as a result of higher temperature reduction was also found to have a considerable effect. In comparison, microstructural differences, such as the foam architecture (lamellar vs foam-like) or the cell size have smaller or negligible effect on the mechanical response. We found that buckling of cell walls is responsible for the non-linear elasticity and the superelasticity up to high strains. The recoverability and energy adsorption depend on density with the latter exhibiting a minimum due to the interplay between wall fracture and wall friction during deformation. These findings suggest that an improvement in the mechanical properties of porous graphene networks significantly depend on the engineering of the flake size that controls the properties of the cell walls.

## Additional Information

**How to cite this article**: Ni, N. *et al.* Understanding Mechanical Response of Elastomeric Graphene Networks. *Sci. Rep.* **5**, 13712; doi: 10.1038/srep13712 (2015).

## Supplementary Material

Supplementary Information

Supplementary Movie S1

Supplementary Movie S2
