# Supplementary material for: Understanding Mechanical Response of Elastomeric Graphene Networks
Source: Sci Rep. 2015 Sep 8;5:13712. doi: 10.1038/srep13712 (PMC4562249; doi:10.1038/srep13712)
Supplement: Supplementary Information [file srep13712-s1.doc]

**Supplementary Information**

**Understanding Mechanical Response of Elastomeric Graphene Networks**

Na Ni^1^*, Suelen Barg^1-^, Esther Garcia-Tunon^1^, Felipe Macul Perez^1^, Miriam Miranda^1^, Cong Lu^2^, Cecilia Mattevi^2^, Eduardo Saiz^1^

^1^Centre for Advanced Structural Ceramics, Department of Materials, Imperial College London, London SW7 2AZ, UK

^2^Department of Materials, Imperial College London, London SW7 2AZ, UK

- Now at: Materials Science Centre, School of Materials, University of Manchester, M1 7HS, UK

Preparation of the GO-sus

Various concentrations (2-20 mg/ml) of GO-sus were obtained by adding organic additives (PVA : sucrose in a 1:1 fixed weight ratio, PVA in the format of 10% aqueous solution) in aqueous GO solution, the GO: total additives ratio was kept as 1:1 in weight. The exact quantities of GO, PVA and sucrose in the GO suspensions can vary depending on the concentration of the starting concentrated GO solution as well as the quantity of the GO-additive suspension to prepare. An example is given below: if we use 10 ml of a concentrated GO solution that has a concentration of 20 mg/ml, we will add 100 mg sucrose and 100 mg PVA (in 10% water solution) so that the weight ratio between GO and total additives is 1:1. Then we dilute the suspension to 40 ml if we desire to obtain a final GO-additive suspension with a concentration of 5 mg/ml.

In-situ SEM of microstructural changes of the porous structures during compression

The microstructural changes of the porous structure during compression were investigated by by in-situ SEM and the results are shown in Figure 6 in the main manuscript and supplementary movie S1 (for the lamella structure) and S2 (for the foam-like structure). 6× in the movie S1 and 8× in S2 indicate the play speed.

Construction of the energy-absorption diagrams for the porous networks

These diagrams predict the highest achievable energy absorption for a given type of materials of certain densities at given conditions (allowable stress and expected strain-rates). The energy-absorption diagrams are constructed from stress-strain curves as shown in Figure 5a of the main manuscript. Firstly, at a fixed strain rate (0.001 s^-1^), the absorbed energy at each peak stress σ_p_ is plotted against σ_p_ for a series of samples with different densities, normalized by the estimated modulus of the cell wall in our r-GO-PNs (~ 10 GPa) (Figure S4). An envelope that just touches each curve can be constructed. This envelope represents the optimum adsorption capability for the material that will depend on its density. The data is replotted on the same axes (*W/E_s_* vs. *σ_p_/E_s_*) and the density points along the envelope can be marked. As the result, the maximum energy absorption is defined for a particular σ_p_ at the given strain rate, and the optimum density to be used can be estimated (the density that gives the σ_p_ along the envelope). The procedure can be carried out for the same type of structure from stress-strain curves obtained at different strain rates (e.g. the lamella structure at the two strain rates of 0.001 and 1 s^-1^ in Figure 10a of the main paper). The density points with similar densities at the two different envelopes for different strain rates are connected, giving a family of lines of constant density.


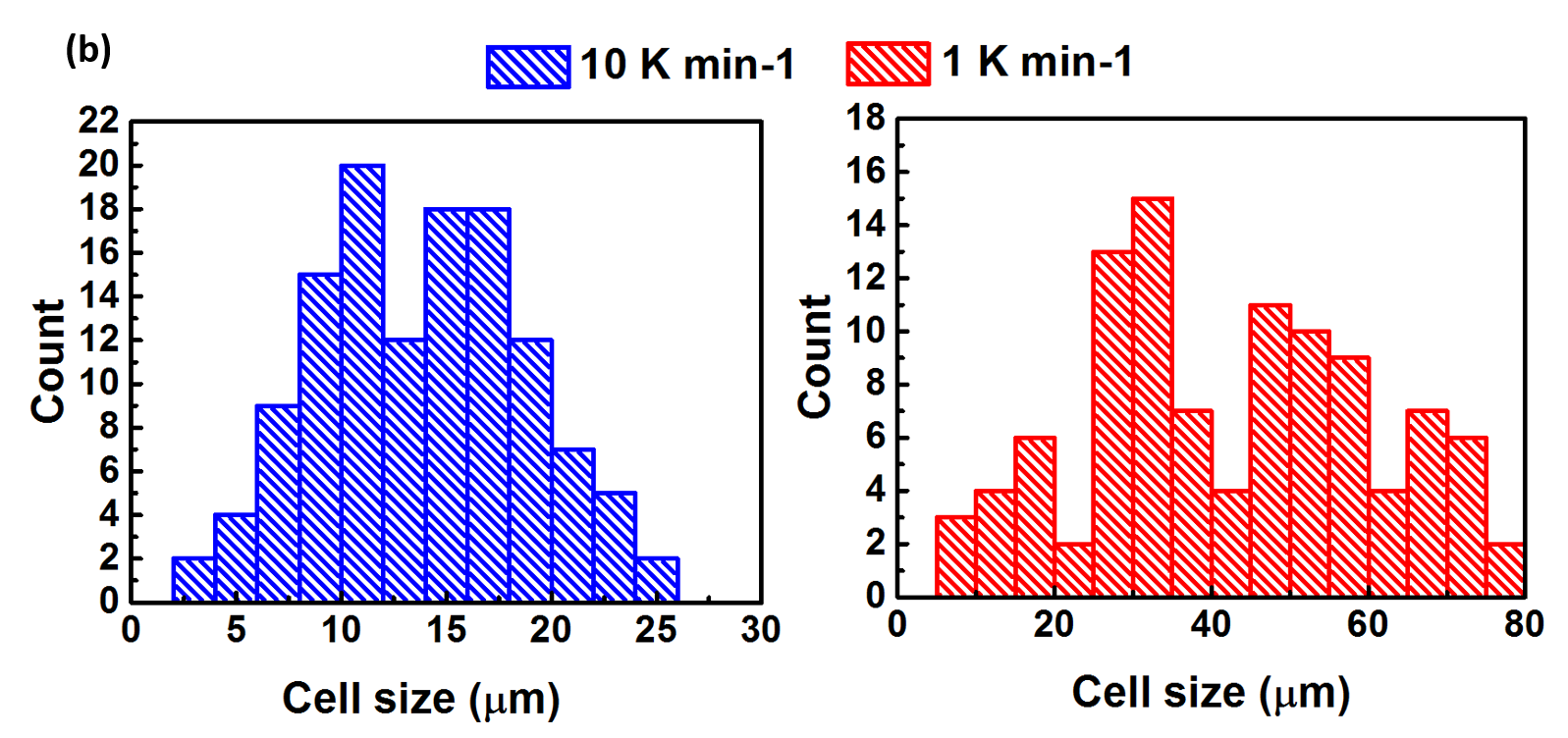


**(a)**

**Figure S1 (a) Cell size of a typical GO-PN prepared at lower freezing speed of 1 K min^-1^.**

Figure S2 Conductivity of rGO-PNs produced using GO flakes of different sizes.

Figure S3 Stress relaxation of a typical r-GO-PN at different stain levels.

Figure S4 (a) Calculation of absorbed energy *W* for each specific peak stress *σ_p_* from the stress-strain curve. (b) Plot of normalized energy vs. normalized peak stress for r-GO-PNs of different density tested at a strain rate of 0.001 s^-1^.
